# Bibliometric analysis of 25 years of research and publications in neuro-oncology in Pakistan: Trends and future directions

**DOI:** 10.12669/pjms.41.13(PINS-NNOS).13448

**Published:** 2025-12

**Authors:** Haseeb Mehmood Qadri, Arham Amir Khawaja, Muhammad Faaiq Ali, Qurat Ul Ain, Arooj Kiran, Hafiz Muhammad Ahtisam Idrees, Syed Haider Hassan, Eeman Afroz, Meer Ahmed, Syed Ather Enam, Asif Bashir

**Affiliations:** 1 Haseeb Mehmood Qadri, MBBS. Postgraduate Resident, Neurosurgery, Punjab Institute of Neurosciences, Lahore, Pakistan; 2 Arham Amir Khawaja, MBBS. Postgraduate Resident, General Surgery and Surgical Oncology, Shaikh Zayed Medical Complex, Lahore, Pakistan; 3 Muhammad Faaiq Ali, Aga Khan University, Karachi, Pakistan; 4 Qurat Ul Ain, Fatima Jinnah Medical University, Lahore, Pakistan; 5 Arooj Kiran, MBBS. Postgraduate Resident, Neurosurgery, Punjab Institute of Neurosciences, Lahore, Pakistan; 6 Hafiz Muhammad Ahtisam Idrees, Ameer-Ud-Din Medical College, Lahore, Pakistan; 7 Syed Haider Hassan, Department of Neurosurgery, Punjab Institute of Neurosciences, Lahore, Pakistan; 8 Eeman Afroz, Allama Iqbal Medical College, Lahore, Pakistan; 9 Meer Ahmed, Mekran Medical College, Turbat, Pakistan; 10.Syed Ather Enam, MD, PhD, FRCSI, FRCSC, FRCSG, FACS. Professor of Neurosurgery, Aga Khan University, Karachi, Pakistan; 11.Asif Bashir, MBBS, FCPS. Professor of Neurosurgery, Punjab Institute of Neurosciences, Lahore, Pakistan

**Keywords:** Brain, Bibliometrics, Developing countries, Neoplasms, Neuro-oncology, Pakistan, Spinal Cord

## Abstract

**Background and Objective:**

Neoplastic lesions of the brain and spinal cord have been on a steady rise in low- and middle-income countries. In recent years, there has been an uprise in neuro-oncology encompassing surgery, oncology, radiology, infectious diseases, and artificial intelligence. However, a comprehensive bibliometric analysis in this field is scarce. The objective of the study was to analyze neuro-oncology publications from Pakistan and quantify the contributions of Pakistani individuals and institutions.

**Methodology:**

A bibliometric analysis of all neuro-oncology research conducted by Pakistani authors or institutions over the last 25 years (2000–2024) was performed using PubMed Central. All relevant case reports, case series, original articles, and review articles were included. The studies conducted in non-Pakistani institutions and collaborative studies were excluded. The data were stratified according to predefined keywords. A total of 210 articles were included in the final analysis.

**Results:**

Among provinces, Sindh contributed the most publications 66.67%(152), followed by Punjab 16.67%(38). Aga Khan University leads from the front with 132 publications. Publications ranged from original articles (37.4%) to review articles (31.1%). Gliomas were among the most common tumours discussed (25.8%). The Journal of Pakistan Medical Association was the most significant contributor (34.7%), followed by Surgical Neurology International (7.14%). Maximum number of studies (53) were published in the year 2024.

**Conclusion:**

An exponential rise in publications in the year 2024 compared to previous years signifies the growing perception of neuro-oncology. However, the dominance of a single institution underscores the need for broader research development across Pakistan.

## INTRODUCTION

Neuro-oncology is a multidisciplinary field dedicated to the study, diagnosis, and management of tumours within the central nervous system (CNS). This domain encompasses primary tumours—such as glioblastomas, meningiomas, and low-grade gliomas—as well as secondary metastatic lesions arising from cancers in other organs.^1^ Globally, advancements in imaging, molecular diagnostics, and targeted therapeutics have transformed clinical outcomes, with regions like North America and Europe boasting robust epidemiological data. For example, the United States reports an average annual age-adjusted incidence rate of 24.83 per 100,000 for both malignant and non-malignant brain and CNS tumours.^2^ In Pakistan, however, neuro-oncology remains relatively underexplored despite a likely substantial burden of CNS tumours and a promising potential for clinical and research advancements, which include aspects of genetics and quality of life in these patients.^3^

A glaring gap in the current literature is the absence of stratified, national data on CNS tumours in Pakistan. No comprehensive registry or systematic record-keeping exists to quantify trends, growth, or regression in the incidence and management of these tumours. Previous analyses have shown that Pakistani neurosurgeons publish an average of only ten PubMed-indexed papers per year, with very few clinical trials, systematic reviews, or basic science studies in this field.^3^ Furthermore, research contributions are markedly concentrated, with limited output from the northern regions of the country. Although bibliometric analyses of neurosurgical research in Pakistan have been conducted, a dedicated quantitative evaluation focusing specifically on neuro-oncology remains absent.^4^ Additional challenges, such as gender disparities in academic authorship—where female-led teams, despite increasing diversity, lag behind their male counterparts—underscore the need for a more comprehensive assessment of the neuro-oncology literature in Pakistan.^5^

To address these critical gaps, our study aims to quantitatively analyze the neuro-oncology contributions made by Pakistani professionals and institutions. By reviewing existing body of scientific literature, we intend to evaluate academic output across various regions, institutional affiliations, and individual contributors. This bibliometric analysis seeks not only to delineate current research trends but also to highlight disparities in scholarly productivity and identify areas where focused improvement is needed. Moreover, by mapping the research landscape, we hope to establish a benchmark for future studies and foster the development of targeted initiatives that enhance both the quality and quantity of neuro-oncology research in Pakistan. Ultimately, this work aspires to contribute to better data-driven strategies that can improve patient care and outcomes in a region where the clinical and academic needs in neuro-oncology are still largely unmet.^6^

## METHODOLOGY

A bibliometric analysis was conducted to investigate the publication trends in neuro-oncology research in Pakistan over the past 25 years. We searched PubMed and indexed Pakistani journals manually for peer-reviewed articles published between 1st January 2000 to 31st December 2024. We preferred PubMed over other databases and chose it as the only target database due to its vast coverage of peer-reviewed biomedical journals, avoiding the inclusion of non-authentic sources and decreasing redundancy from multiple databases.

Specific predefined combination of keywords using Boolean operators (AND/OR) were employed to capture a broad range of articles related to neuro-oncology in Pakistan which are as follows: “Brain Tumor Pakistan”, “Brain SOL Pakistan”, “Brain SOL AND Pakistan”, “Brain tumour AND Pakistan”, “Cranial tumour AND Pakistan”, “Pituitary Tumor Pakistan”, “Gliomas Pakistan”, “Meningioma Pakistan”, “Cerebellopontine angle SOL Pakistan”, “Deep Seated SOLs Pakistan”, “Pineal Gland SOLs Pakistan”, “Olfactory region SOLs Pakistan”, “Interventricular SOLs Pakistan”, “Craniopharyngioma Pakistan”, “Third/Fourth Ventricles of Pakistan”, “Spine Tumor Pakistan”, “Spinal Tumor Pakistan”, “Spinal SOL AND Pakistan”, “Spine SOL AND Pakistan”, “Spinal tumour AND Pakistan”, “Spine tumour AND Pakistan”, “Vertebral tumours AND Pakistan”. The word ‘tumour’ was interchanged with ‘tumor’ also and the searches were repeated. We incorporated keywords related to Pakistan’s regions, cities and institutions to refine our research during the mentioned time frame. To minimize duplication, remove selection bias and maximize relevance, HMQ and AAK cross-checked the retrieved literature with respect to article title and date of publication.

### Inclusion criteria:

The following inclusion criteria were applied to ensure comprehensive neuro-oncology research:

- Authors were affiliated with Pakistani institutions and working in those institutions at the time the study was conducted, and research conducted exclusively in Pakistan within the specified timeframe.
- All the neuro-oncology articles with the following domains: surgery in neuro-oncology, neurovascular, neuro anesthesia, neuro-radiology, interventional radiology, neuro-pathology, infections in neuro-oncology, radiotherapy in neuro-oncology, gamma knife surgery, chemotherapy, genetic studies, innovation, nanotechnology, artificial intelligence, nursing care in neuro-oncology and rehabilitation in neuro-oncology were included.
- Various research designs, including case reports, case series, letter to editor, short communication, case-cohort studies, cohort studies, cross-sectional studies and clinical trials were recruited.

### Exclusion criteria:

- Studies authored by non-Pakistani researchers or those conducted outside Pakistan or collaborative studies in which Pakistani coauthors were present.
- Cadaveric studies, animal studies, conference abstracts, and non-scientific items (e.g. newspaper articles, roll calls).

### Data extraction and analysis:

The results from the mentioned database were scrutinized to extract relevant information, including study location and timeframe, type of tumour investigated, article type and journal of publication. We created a Google form to systematically enter the extracted data. Subsequently, the data was analyzed using Microsoft Word and Excel (Microsoft Office 365; Microsoft Corporation, Redmond, WA, USA); IBM SPSS software version 24 (IBM Corporation, Armonk, NY, USA) to organize and categorize the data, identify trends and patterns and generate visual representations of the findings in the form of graphs and charts and present data distribution in percentages. To summarize, raw data from each included article were extracted and entered using Google Forms (Google LLC, Mountain View, CA, USA). Subsequently, the compiled dataset was analyzed and organized using Microsoft Word and Microsoft Excel (Microsoft Office 365; Microsoft Corporation, Redmond, WA, USA). Statistical analysis and descriptive statistics were performed using IBM SPSS Statistics for Windows, version 24.0 (IBM Corporation, Armonk, NY, USA) Final data, satisfying the inclusion and exclusion criteria equilibrated to 210 articles. The data has been compiled accordingly to make it representable. However, the citations of all included studies are available in a separate supplementary table, which can be made available upon request.

## RESULTS

Most publications were from Sindh Province 152 (66.67%), followed by Punjab Province, 38 (16.67%) and Islamabad/Federal, 20 (8.77%) (Table-I). By city, Karachi accounted for 152 (66.67%) of the contributions, encompassing all publications from Sindh, while Lahore contributed to 28 (12.28%) and Islamabad to 20 (8.77%), together representing the majority of neuro-oncology publications. (Table-II).

**Table-I:** Province-wise distribution of included Neuro-oncology publications (N=228). Note: N represents the total number of author–affiliation instances, which exceeds the number of studies (n=210) because publications with multiple authors from different provinces contributed more than one entry.

Provinces	Frequency (n)	Percentage (n/N)
Sindh	152	66.67%
Punjab	38	16.67%
Islamabad/Federal	20	8.77%
Khyber Pakhtunkhwa	12	5.26%
Nationwide	5	2.19%
Baluchistan	1	0.44%
Total (N)	228	100%

**Table-II:** City-wise distribution of included Neuro-oncology publications (N = 228). Note: N represents the total number of author–affiliation instances, which exceeds the number of studies (n = 210) because publications with multiple authors from different cities contributed more than one entry.

City	Frequency (n)	Percentage (n/N)
Karachi	152	66.67%
Lahore	28	12.28%
Islamabad	20	8.77%
Peshawar	10	4.39%
Rawalpindi	9	3.95%
Mardan	2	0.88%
Miscellaneous*	7	3.07%
Total (N)	228	100%

* Sargodha, Quetta, Larkana, Faisalabad, Dera Ghazi Khan, Bahawalpur, Abbottabad.

Strikingly, most of the publications were contributed by Aga Khan University, Karachi, Pakistan, comprising 46.81% of the total publications. To a lesser extent, 6.03% of contributions were from Shaukat Khanum Memorial Cancer Hospital and Research Centre, Lahore, Pakistan, 3.55% contributions were from Dow University of Health Sciences, Karachi, and 2.48% contributions were from Jinnah Postgraduate Medical Centre, Karachi (Table-III). Additionally, 57 (27.14%) were from public sector institution, 153 (72.85%) were from private sector institution (Table-III).

**Table-III:** Institute-wise distribution of included Neuro-oncology publications (N = 282). Note: N represents the total number of author–affiliation instances, which exceeds the number of studies (n = 210) because publications with multiple authors from different institutions contributed more than one entry.

Institutions	Frequency (n)	Percentage (n/N)
Aga Khan University, Karachi, Pakistan	132	46.81%
Shaukat Khanum Memorial Cancer Hospital and Research Centre, Lahore, Pakistan	17	6.03%
Dow University of Health Sciences, Karachi, Pakistan	10	3.55%
Jinnah Postgraduate Medical Centre, Karachi, Pakistan	8	2.84%
Liaquat National Hospital, Karachi, Pakistan	8	2.84%
COMSATS University, Islamabad, Pakistan	7	2.48%
Punjab Institute of Neurosciences (PINS), Lahore, Pakistan	7	2.48%
Rehman Medical Institute, Peshawar, Pakistan	6	2.13%
Civil Hospital, Karachi, Pakistan	6	2.13%
Islamic International Medical College, Rawalpindi, Pakistan	5	1.77%
Khyber Medical College/Khyber Teaching Hospital, Peshawar, Pakistan	5	1.77%
Pakistan Institute of Medical Sciences (PIMS), Islamabad, Pakistan	5	1.77%
National University of Sciences and Technology (NUST), Islamabad, Pakistan	4	1.42%
Shifa International Hospital, Islamabad, Pakistan	4	1.42%
Liaquat University of Medical and Health Sciences, Jamshoro, Pakistan	4	1.42%
Shaheed Mohtarma Benazir Bhutto Medical College Lyari, Karachi, Pakistan	3	1.06%
Combined Military Hospital, Rawalpindi/Lahore/Peshawar	3	1.06%
King Edward Medical University/Mayo Hospital, Lahore, Pakistan	2	0.71%
Allama Iqbal Medical College / Jinnah Hospital, Lahore, Pakistan	2	0.71%
Ziauddin Medical College, Karachi, Pakistan	2	0.71%
Jinnah Sindh Medical University, Karachi, Pakistan	2	0.71%
University of Peshawar, Peshawar, Pakistan	2	0.71%
Miscellaneous**	37	13.12%
Total (N)	282	100%

** Gambat Institute of Medical Sciences (GIMS), Hayatabad Medical Complex, Nationwide study, Abbasi Shaheed Hospital, Pakistan Institute of Rehabilitation Sciences (PIRS), University of Engineering and Technology (UET) Lahore, Government College University Faisalabad, Institute of Space Technology Islamabad, Lahore University of Management Sciences (LUMS), University of Management and Technology (UMT) Lahore, Ayub Medical Institute and Mardan Medical Complex, Nuclear Medicine Oncology and Radiotherapy Institute (NORI), South City Hospital Karachi, The Indus Hospital Karachi, Bahawalpur Victoria Hospital, Karachi Cancer Registry, Ayub Teaching Hospital Abbottabad, Chandka Medical College, University College of Medicine and Dentistry Lahore, Baqai Medical University Karachi, Pakistan Gamma Knife & Stereotactic Radiosurgery Center, Neurospinal & Medical Institute Karachi, Brain Surgery Clinic Rawalpindi, District Health Quarters Rawalpindi, Holy Family Hospital Rawalpindi, Institute of Radiotherapy and Nuclear Medicine Peshawar, Center for Nuclear Medicine and Radiotherapy Quetta, Jinnah Medical and Dental College Karachi, Ghurkhi Trust and Teaching Hospital Lahore, Lahore General Hospital, Lady Reading Hospital Peshawar, National University of Computer and Emerging Sciences Lahore, Patel Hospital Karachi, Ziauddin Medical University Karachi, Foundation for Advancement of Science and Technology (FAST) Lahore, Isra University Islamabad Campus, Memon Medical Institute Hospital Karachi, and Cancer Foundation Hospital Karachi.

**Fig.1:**
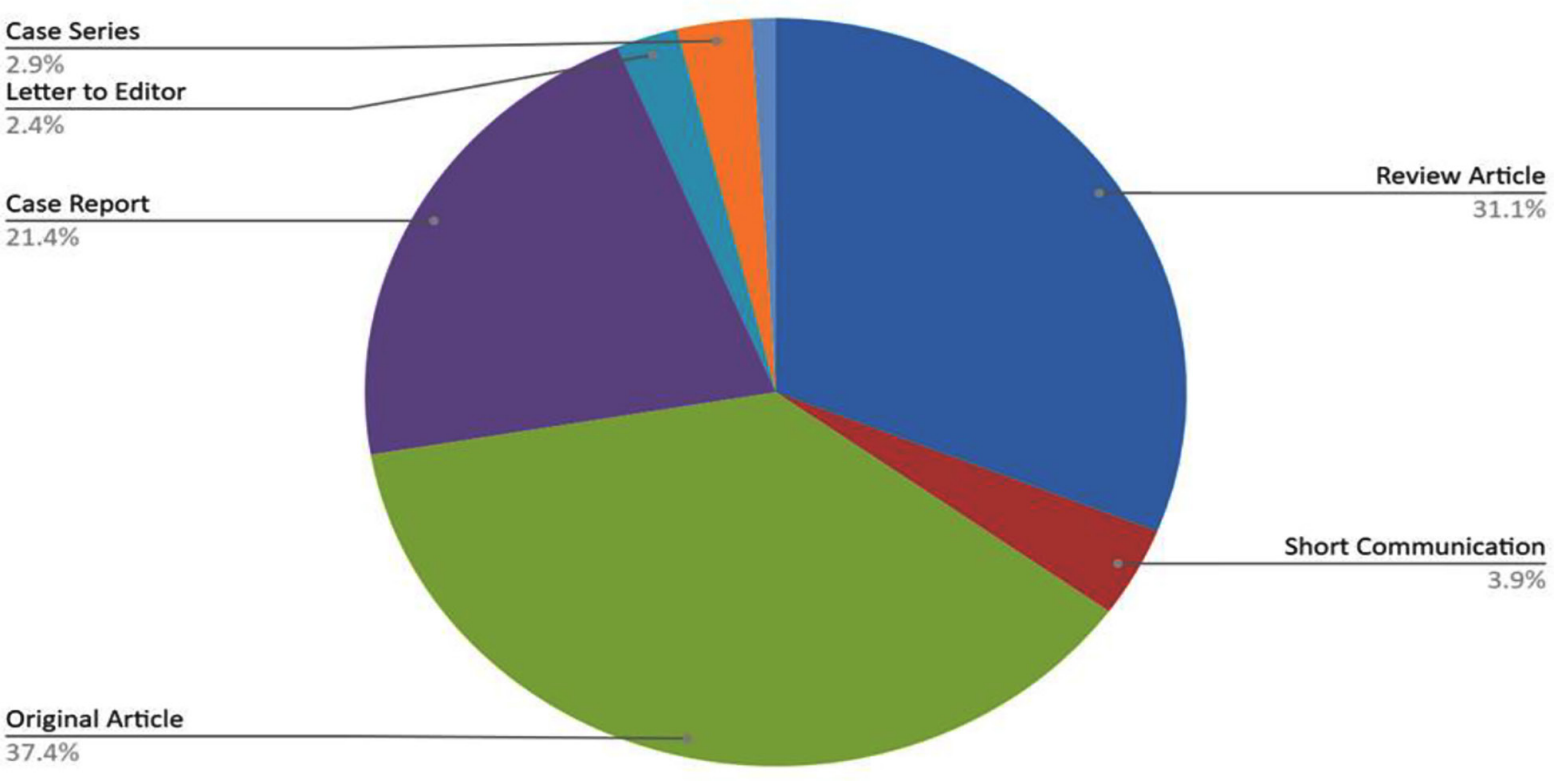
Distribution of included publications based on article type.

Out of the 210 articles isolated, 37.4% were original articles, 31.1% were review articles, and 21.4% consisted of case reports. Short communication, case series and letter to editor corresponded to about 3.9%, 2.9%, and 2.4%, respectively (Fig.1).

**Fig.2:**
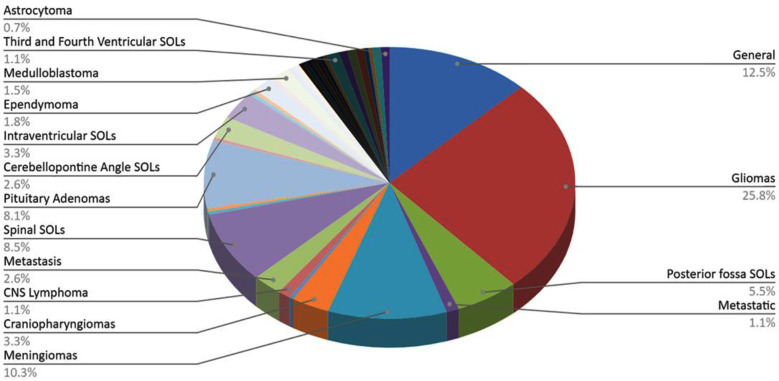
Tumors discussed in the Publications).

A wide range of tumours was discussed in the publications. The most commonly discussed was Gliomas, in 70 publications (25.8%). In about 12.5%, there was a General brain/spine tumour discussion, while Meningiomas were discussed in about 10.3% of the publications (Fig.2).

**Fig.3:**
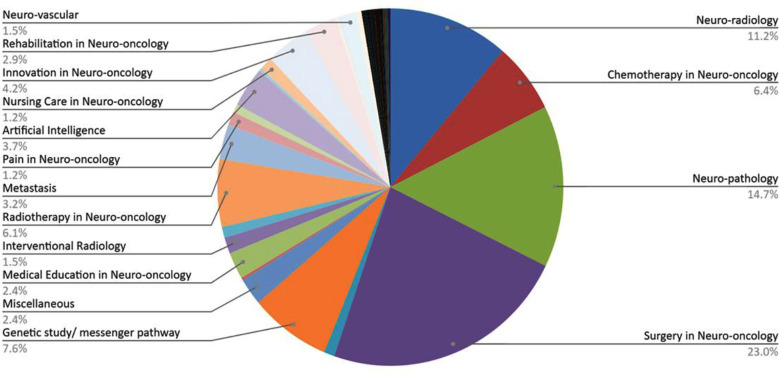
Study Components discussed in the included Neuro-oncology Publications.

Numerous study components were discussed in the publication. Study components in this review refer to the subject areas addressed within the included neuro-oncology publications. Each component represents a focused aspect of neuro-oncology research, clinical practice, or education, ranging from subspecialty discipline, such as neurovascular, neuroanesthesia, neuroradiology, to treatment modalities that include radiotherapy, chemotherapy, gamma knife surgery, research approaches that constitute genetic studies, artificial intelligence, and supportive care, pain management, nutrition, nursing care, rehabilitation, and other specialized or emerging topics relevant to the field. The most common was surgery in neuro-oncology (23.0%), followed by neuro-pathology (14.7%), neuro-radiology (11.2%) and genetic study/messenger pathway (7.6%) (Fig.3).

About 118 (57.00%) of the articles were published in International journals, and 89 (43.00%) were published in Local Journals. About 158 (76.33%) were in open-access journals, 29 (14.01%) were hybrid journals, and 20 (9.66%) were subscription-based journals. The information from the eight journals could not be retrieved. Among the local journals, the Journal of Pakistan Medical Association (JPMA) published the most articles (34.76%), while Surgical Neurology International (7.14%) and Neurosurgical Review (5.24%) published the most articles among the international journals (Table-IV).

**Table-IV:** Journal-wise distribution of included Neuro-oncology Publications (N=210).

Journals	Frequency (n)	Percentage(n/N)
Journal of Pakistan Medical Association (JPMA)	73	34.76%
Surgical Neurology International	15	7.14%
Neurosurgical Review	11	5.24%
Neuro oncology	8	3.81%
Journal of Ayub Medical College Abbottabad (JAMC)	8	3.81%
Pakistan Journal of Medical Sciences (PJMS)	7	3.33%
Asian Journal of Neurosurgery	7	3.33%
Child’s Nervous System	6	2.86%
BMJ Open	5	2.38%
World Neurosurgery	4	1.90%
PLoS One	4	1.90%
The Neuroradiology Journal	4	1.90%
Brain Tumor Research and Treatment	4	1.90%
Future Oncology	3	1.43%
Cureus Journal of Medicine	3	1.43%
Journal of Neuro-Oncology	2	0.95%
BMC Research Notes	2	0.95%
International Journal of Surgical Case Reports	2	0.95%
Asian Pacific Journal of Cancer Prevention	2	0.95%
Journal of Pediatric Hematology/Oncology	2	0.95%
Miscellaneous**	37	17.62%
Total (N)	210	100%

** Clinical Neurology and Neurosurgery, Journal of Clinical Neuroscience, BMJ Paediatrics Open, Journal of Cancer and Allied Specialties, Digital Health (Saga Journal), World Neurosurgery X, Molecular Biology Reports, Natural Product Research, Journal of Public Health Research, Frontiers in Psychology, Brain and Spine, Pharmaceutics, Molecular Genetics and Genomics, Molecules, World Journal of Surgical Oncology, Current Medical Research & Opinion, Revista da Associação Médica Brasileira, Medical Engineering & Physics, Psychooncology, Journal of Patient-Reported Outcomes, BMJ Case Reports, Journal of Neurosurgical Sciences, Elsevier's Computers in Biology and Medicine, Journal of Digital Imaging, Biomed Pharmacother, Computerized Medical Imaging and Graphics, Physics in Medicine & Biology, Scientific Reports, International Journal of Biomedical Imaging, Journal of Family Medicine and Primary Care, Bioscience Reports, Current Medical Science, Neuropathology, Acta Radiologica Short Reports, Annals of Diagnostic Pathology, International Journal of Surgical Pathology, Microscopy Research and Technique, Biomed Central (BMC), Journal of Radiosurgery and SBRT, BMC (Journal of Medical Case Reports), Journal of Pediatric Hematology and Oncology, Journal of College of Physicians and Surgeons (JCPSP), British Journal of Neurosurgery.

The mean number of authors was 5.245, with a standard deviation of 3.01. The authors; Nida Zahid (3.3%), Mohammad Hamza Bajwa (2.9%), Ummey Hani (2.9%), Fauzan Alam Hashmi (2.9%), Mujtaba Khalil (1.9%), Hafiza Fatima Aziz (1.9%), Mashal Murad Shah (1.9%), contributed as first authors. Muhammad Shahzad Shamim (19.0%), Syed Ather Enam (12.7%), and Naureen Mushtaq (2.4%) contributed as the last authors in the publications.

There was a roughly equal distribution of publications in different months, with most being in November (32), March (25), and September (22), corresponding to about 15.31%, 11.96%, and 10.53%, respectively.

**Fig.4:**
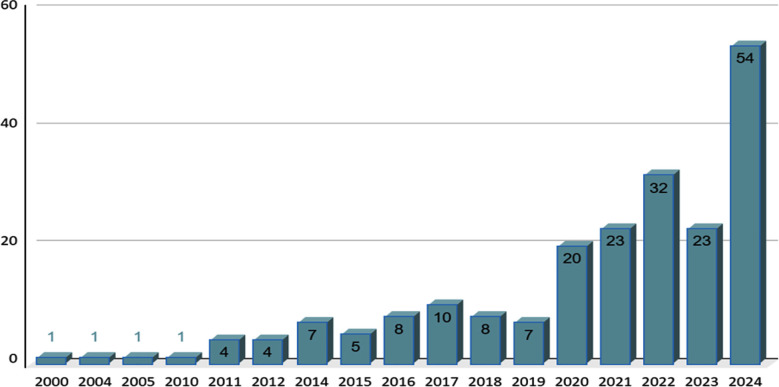
Yearly distribution of Neuro-oncology Publications from Pakistan over the last 25 years (N=210).

A general exponential trend was observed over the last 25 years in neuro-oncology, with a maximum number of publications observed in the year 2024 (53) (Fig.4). About 41 publications could be scrutinized from the time of submission of the article till publication. The mean time was about 71.56 ± 69.92 days.

## DISCUSSION

In 25 years, neuro-oncology-focused research has exponentially grown in the country. A positive trend was observed from 2019 to 2020, where an increase of 13 publications was seen. This number has since gone upward, and last year, in 2024, 54 papers were published. In contrast, only 4 publications were present from 2000-2010, and 53 papers were published from 2000-2020 on neuro-oncology. Though Pakistan’s contribution to neurosurgery research has been low in comparison to developed nations, publication rates have risen from 2.4% to 4.1% from 2017 to 2020, and it has become the fourth most contributing country in LMICs.^7^ In comparison, Ethiopia; another LMIC has published only 9 papers in neuro-oncology from 2000-2021.^8^This represents the acknowledgement of the pivotal role of research in healthcare and medical education by Pakistanis and a positive attitude towards publishing.

Geographically, the number of studies has been significantly skewed towards the province of Sindh, having the highest number of publications at 66.67% (n=152), followed by Punjab at 16.67% (n=38), and the third biggest contributor was Islamabad/Federal Territory with 8.77% (n= 20). The skew is caused by Sindh being the home of the highest publishing centre, Aga Khan University, Karachi, for which reasons have been discussed further in the text.

Similarly, Karachi, the capital of Sindh, has had the greatest number of publications, 66.67% in the context of city-wise distribution, signifying that all publication output from Sindh was only from Karachi. This warrants the need to promote research culture in centres situated in other cities of Sindh and tackle the regional disparity in the province. This would not only develop academic prowess in professionals in the field but will also contribute to efficient healthcare and increased availability of context-specific data that can be utilized in a better understanding of disease pathologies.

Moreover, it was also noted that the cities with the most publications were commonly the capitals of the Provinces or the Federal Capital, with Lahore being second at 12.28% (n=20), followed by Islamabad at 8.77% (n=20), and Peshawar at 4.39% (n=10). The authors believe there might be two factors associated with the finding: Firstly, uneven geographical distribution of research-oriented individuals and resources to produce quality research. Secondly, the preference of the general population to acquire healthcare at larger centres for neuro-oncology.

Aga Khan University, Karachi, has had the highest number of publications at 46.81% (n=132) followed by Shaukat Khanum Memorial Cancer Hospital and Research Center, Lahore Pakistan, having 6.03% (n= 17) of share. Dow University of Health Sciences, Karachi stands third with Jinnah Postgraduate Medical Centre, Karachi being fourth with 3.55% (n=10) and 2.84%(n=8), respectively. Institutions in Karachi have a greater share of publications, for which the factors could be explored in future studies. The possible factors could be a larger population, a wider exposure and interaction with international faculty and alumni. Furthermore, it is important to mention that the top two centres are Not-for-Profit organizations with international and national funding, whereas most other institutions have limited Government funding. These centres not only have large funding but also academic faculty that have trained in the West and are competent researchers.

The published works had a wide variety of topics ranging from surgical aspects of neuro-oncology (23%) to medical education in neuro-oncology (2.45%). Larger proportions were shared by surgery in neuro-oncology, neuro-pathology (14.75%), neuro-radiology (11.2%) and chemotherapy in neuro-oncology (6.4%). Other notable works entailed artificial intelligence (3.7%), radiotherapy (6.1%) and genetic studies (7.6%). Recently, Artificial Intelligence (AI) has been incorporated into patient care extensively, from pre-operative planning to post-operative care.^9^ AI can help cope with the challenges of resource scarcity in a resource-limited setting such as Pakistan.^10^ Therefore, it is essential to promote further data-supported evidence that could be employed to assess feasibility and plan the use of AI in patient care. Since 2016, WHO has added molecular and genetic markers along with histological characteristics in labelling tumors, which was further updated in the 2021 CNS Tumor Classification.^11^ Thus, the importance of genetic and molecular studies cannot be left unnoticed. It is imperative to keep up with the constantly evolving guidelines to ensure a tailored patient care experience that can benefit patients. In Pakistan’s context, developing research potential in the role of AI and genetic/molecular studies in neurosurgery face the great challenge of acquiring sufficient funds, adequate equipment and an expert workforce in the field.

The most common types from Pakistan that were discussed in the publications were Gliomas (25.8%), Meningiomas (10.3%) and Pituitary Adenomas (8.1%). This complements the global trends of neuro-oncology as Gliomas have been one of the most studied CNS tumour types in recent years. High-grade gliomas, till this date, have a poor prognosis with survival rates as low as 22-18 months^12^, which marks the need for further research to improve outcomes and Quality of Life indicators of diagnosed patients.

Mostly, the work coming out of Pakistan in neuro-oncology has been Original Articles (37.45%), Review Articles (31.1%) and Case Reports (21.4%). Having a fairly distributed type of research output is a positive finding and expresses an effort to achieve a well-rounded research environment in the country. Such an approach allows to obtaining of holistic evidence where original articles and case reports are means to obtain grass-root and contextual data, whereas review articles aim to compile and comment on existing evidence and strive to achieve consensus on treatment strategies. This shows that Pakistan is contributing ground-level data for further research to the international research fraternity as much as utilizing existing data to produce novel research publications.

The majority of articles were published in JPMA, a national journal with 34.76% (n=73), followed by Surgical Neurology International 7.14% (n=15) and Neurosurgical Review 5.24% (n=11), both being international journals. About 57.00% (n=118) of the articles were published in international journals, and 43.00% (n=89) were published in National Journals. Around 21% (n=44) of articles were accepted into journals with impact factors greater than 2.0, according to the recent ratings. The disparity between a greater number of publications in open-access journals and a low number in high-impact-factor journals is a result of multiple reasons. Namely, the high fees charged by journals^13^, strict scrutiny of publication content and a lack of high-quality research restrict researchers from publishing in respected and impactful journals.

### Limitations:

Certain limitations need to be elaborated. We only used data available to us on PubMed Central. Articles which were subscription-based and from other databases such as Cochrane, Embase, Wiley, and Google Scholars were not scrutinized. articles. It remains a possibility that certain articles are published by journals indexed in PubMed but do not result in being indexed in PubMed individually. Additionally, we excluded all collaboration studies, which were a significant part of Pakistani co-author contributions.

## CONCLUSION

Academia in Pakistan are consistently making strides to publish works in the field of neuro-oncology as seen in the increasing number of publications throughout the years. An exponential rise in publications in the year 2024 compared to previous years signifies the growing perception of neuro-oncology. However, the dominance of a single institution underscores the need for broader research development across Pakistan. Nevertheless, a lag has been identified in the content and quality of publications and concentrated representation by one city and province, which needs to be tackled. Support from the institutions and international entities in terms of funds, workforce and resources is essential to overcome the said challenges to guarantee equity and quality in healthcare for the people of Pakistan.

### Recommendations:

The disparity mostly exists due to a lack of awareness and training in the field of neuro-oncology, especially among the healthcare professionals of Pakistan. To address the disparity in Neuro-oncology, the institutes of Punjab, Khyber Pakhtunkhwa and Baluchistan must prioritize establishing dedicated Neuro-oncology research centres in collaboration with existing research centres in Karachi to share expertise and resources, and these units should focus on documentation and publication. Proper funding to the institutions of other provinces must be allocated to assist the Neuro-oncology research. Neuro-oncology research awareness should be provided among the healthcare professionals of different institutes in Pakistan.

Clinicians and researchers should play a leadership role by providing mentorship to the under-represented provinces to address the disparities in neuro-oncology research. Training programs and workshops must be organized for the clinicians to build their research skills, which include research methodology, data collection, and analysis. Lastly, a national platform for the registry of Neuro-oncology cases must be established. The platform should facilitate a centralized database to systematically collect and analyze the data from all the institutions in Pakistan.

### Note:

Final data, satisfying the inclusion and exclusion criteria equilibrated to 210 articles. The data has been compiled accordingly to make it representable. However, the citations of all included studies are available in a separate supplementary table, which can be made available upon request.^14-223^

### Author`s Contribution:

**HMQ:** Concept of the study, Critical review of the manuscript and supervision.

**AAK:** Data acquisition, analysis and interpretation and drafted the manuscript.

**MFA, QUA, AK, HMAI, SHH, EA and MA:** Data interpretation and drafted the manuscript.

**SAE and AB:** Data interpretation and critically reviewed the manuscript.

All the authors have read and approved the final manuscript and are responsible and accountable for the accuracy and integrity of the work.

## References

[1] Lamba N, Wen PY, Aizer AA (2021). Epidemiology of brain metastases and leptomeningeal disease. Neuro Oncol.

[2] Ostrom QT, Price M, Neff C, Cioffi G, Waite KA, Kruchko C (2023). CBTRUS statistical report:Primary brain and other central nervous system tumors diagnosed in the United States in 2016–2020. Neuro Oncol.

[3] Zahid N, Martins RS, Brown N, Zahid W, Azam I, Hassan A (2023). Psychosocial factors influencing quality of life in patients with primary brain tumors in Pakistan:An analytical cross-sectional study. BMC Res Notes.

[4] Waqas M, Siddiqui UT, Shamim MS (2019). Follow-up bibliometric analysis of neurosurgical publications from Pakistan and institutional comparison with other countries using h-index and i-10 index. Asian J Neurosurg.

[5] Garg K, Chaurasia B, Gienapp AJ, Splavski B, Arnautovic KI (2023). Bibliometric analysis of 6 major European neurosurgical publications from 2011–2020 (part 3):A comparative metrics review. Acta Inform Med.

[6] Behmer Hansen RT, Behmer Hansen RA, Gold JL, Batchu S, Lozada RD, Palma SD (2022). Neuro-oncology authorship trends in gender since 1944:A systematic review of 14,020 articles from five top-tier academic journals. J Neurosurg.

[7] Abdi H, Wang Z, Ham EI, Laeke T, Park KB, Negida A (2022). Neurosurgery research output in Ethiopia:A scoping review. World Neurosurg.

[8] Cannizzaro D, Safa A, Bisoglio A, Jelmoni AJM, Zaed I, Tropeano MP (2022). Second footprint of reports from low- and low- to middle-income countries in the neurosurgical data:A study from 2018–2020 compared with data from 2015–2017. World Neurosurg.

[9] Noh SH, Cho PG, Kim KN, Kim SH, Shin DA (2023). Artificial intelligence for neurosurgery:Current state and future directions. J Korean Neurosurg Soc.

[10] Awuah WA, Kalmanovich J, Mehta A, Huang H, Yarlagadda R, Kundu M (2023). Harnessing artificial intelligence to bridge the neurosurgery gap in low-income and middle-income countries. Postgrad Med J.

[11] Louis DN, Perry A, Wesseling P, Brat DJ, Cree IA, Figarella-Branger D (2021). The 2021 WHO classification of tumors of the central nervous system:A summary. Neuro Oncol.

[12] Wen PY, Kesari S (2008). Malignant gliomas in adults. N Engl J Med.

[13] Jubran JH, Scherschinski L, Benner D, Park MT, Rhodenhiser EG, Ibrahim S (2023). Publication speed across neurosurgery journals:A bibliometric analysis. World Neurosurg.

